# On economists as policy advisors with applications to Switzerland

**DOI:** 10.1186/s41937-017-0009-4

**Published:** 2018-01-25

**Authors:** Aymo Brunetti

**Affiliations:** Department of Economics and Center for Regional Economic Development (CRED), University of Bern, Bern, Switzerland

## Abstract

This paper analyses the role of economists in advising political agents. Based on the experience in the recent financial crisis, it starts with the important role of expectations management. Economists should make clear that precise forecasts are not possible but that application of economic principles and careful analysis of data and historical events can substantially improve political decisions. In order to be effective, economic advisors have to be aware of the first best as well as of the political feasibility of proposals. Efficiency is the obvious benchmark, but a large part of policy advice is about finding the least inefficient of the feasible alternatives. The paper argues that a crucial precondition for being an effective policy advisor are communication skills; academics who become policy advisors should not try to impress their peers but rather translate insights in a language that is understandable for educated laypeople. The paper then looks at the special situation of economic policy advice in the Swiss direct democracy before concluding with a summary of the most important preconditions for being a successful policy advisor.

## Introduction

“Why did no one see it coming?” Queen Elizabeth II asked her embarrassed hosts when hearing about the effects of the global financial crisis that had recently broken out during a visit at the London School of Economics in November 2008. This frequently cited question summarises one possible interpretation of economists’ role in policy consulting: When the crisis struck, neither did economists have a useful forecast to offer nor could their models assess the effects of this financial earthquake on the global economy. However, there is a second diametrically opposed interpretation of the same event. Economists analysed the penultimate global financial crisis—the Great Depression of the 1930s—in such a thorough manner that they were able to tell politicians which economic policy measures had to be taken in order to prevent a recurrence of such a crisis next time. In fact, the combination of extraordinary monetary policy measures, support of banks and fiscal reactions probably prevented the outbreak of a second great depression in 2008. This economic policy reaction was largely rooted in the scientific analysis of the failures in policy advice and measures taken in the 1930s.

This paper is organised as follows: The “Expectations vs. deliverables” section argues how important expectations management is for economic policy advisors. The “Efficiency vs. feasibility: goals of economists’ policy advice” section discusses the goals of economists’ policy advice, and the “Communication is crucial” section is on the crucial importance role of communication for successful policy advice. The “Economic policy advising in Switzerland” section turns to the specific situation for policy advisors in Switzerland with its direct-democratic political system, and the “Concluding remarks” section concludes.

## Expectations vs. deliverables

The introductory example shows the importance of having realistic expectations what economic advice is capable to deliver, and what not. Our science has frequently been criticised for being unable to make precise forecasts. However, this demand, also frequently made by many politicians, is far too ambitious. Economic life is too complex, there are too many influencing factors and measurability is too incomplete to expect reliable quantitative forecasts of macroeconomic events. This is not only true for forecasts but is also valid for quantitative evaluations on the effects of economic policy decisions. In my opinion, anyone working in the field of economic policy consulting should categorically resist policymakers’ demands for precise forecasts; sometimes this works, sometimes not. To give a positive example: expectation management concerning quantitative forecasts has been successfully conducted only recently in an important economic policy decision in Switzerland. When at the beginning of the financial crisis in 2008 it became necessary to design economic policy reactions, it was easy to communicate in the face of the extraordinary shock that a precise economic forecast as a basis was not feasible. This insight facilitated the breakthrough of a sensible plan of action. Right from the start a step-by-step procedure for the implementation of fiscal stabilisation measures was chosen. Decisions on each step took into account the latest economic data and therefore depended on continually adjusted economic expectations. Thus, at the outset, it was deliberately decided not to base the whole programme on a supposedly precise quantitative forecast.^1^ In this specific situation, it was possible to explain the infeasibility of such an approach to policymakers, who naturally would have preferred a precise forecast. In less extraordinary cases, this, unfortunately, regularly proves to be much more difficult.

If quantitative forecasts are not possible with sufficient precision, what kind of analysis can economics contribute to economic policy consulting? In my opinion, there are essentially three feasible forms of deliverable input for policy advice. First—often underestimated—economists can get some mileage by contributing simple and measurable facts to the policy debate. Political exponents, who proclaim the end of jobs every time new technology emerges, can for example easily be confronted with the simple fact that employment in Switzerland has continually risen in the last century—despite immense technological progress. Secondly, basic economic concepts can be helpful in convincingly explaining the consequences of economic policy measures. Simple supply and demand curve analysis suffices to show that a minimum wage, which is significantly higher than the equilibrium wage will reduce employment. And thirdly, economic history provides a wealth of material through which one can learn from the results of past economic policy measures. The introductory example of the analysis of economic policy and its shortcomings during the Great Depression impressively demonstrates the immediate value of such ex-post evaluations. It is certainly possible, therefore, to expect reliable estimates on the qualitative effects of economic policy measures from economics.

## Efficiency vs. feasibility: goals of economists’ policy advice

We now turn to the question of what economists aim for when advising policymakers.

Economists are mainly specialists on the allocation of scarce resources. In advising policymakers their most important role, therefore, is to bring the aspect of efficiency into the political discussion.^2^ In an ideal (and rather rare) case, policymakers ask economic advisors how to design policies in order to maximise welfare, i.e. the sum of consumer and producer rents. In this situation, economists will use traditional microeconomic theory and push for market solutions as long as no market failures distort allocative efficiency. In case of clear market failures (i.e. monopolistic power, externalities, public goods and some forms of information asymmetries), economists can—again based on established concepts—suggest the most efficient measures to deal with them. However, much more frequent is the situation where politicians have already decided to intervene on an issue due to non-efficiency goals and the task of the economic advisor is to suggest on the best way to proceed. In this situation, it is not the first best that economists can aim for but rather the least inefficient way to reach the politically defined goal.

In this context, a crucial question emerges for any economist who chooses to leave pure academic work and give policy advice: How should politics be taken into account? Should the economist always insist on propagating the most efficient solutions independently of their feasibility? Or should they limit themselves to the politically achievable? From my point of view, both approaches have their merits and which is selected primarily depends on the institutional role the respective economist assumes. Should the economist be an external observer who comments on economic policy issues as a scientist, it is helpful to state clearly by which approach scarce resources can most efficiently be used without worrying too much about politics. Such statements can serve as general guidelines for the economic policy debate. However, if economists limited themselves to such statements, it would be easy to dismiss their recommendations as purely academic. As soon as economists are more involved institutionally, they also have to consider other options than the first best solution. In such cases—as already alluded to—the politically achievable possibilities are to be analysed choosing the one, which results in the lowest possible degree of inefficiency. In this role as a scientific collaborator or a chief economist of an administration, the main task is to point out economic costs and benefits and—if necessary—propose alternatives. The challenge is to signalise that one is aware of political realities without losing sight of economic efficiency. The added value of economists in a public administration is to point out the economically most efficient solution among the politically achievable possibilities without seeming politically naïve and not to assume the role of a political advisor (others can do so better).

In this respect, a suggestion by Acemoglu and Robinson (2013) seems too ambitious. They argue that advisors should not only consider and propagate the most efficient solution but they should also take into account the effect of suggested policies for future political equilibria. If for example an efficiency-enhancing policy weakens labour unions, this could, in the future, lead to a political equilibrium that inefficiently favours business interests. In my view, this can be an interesting approach for understanding the development of institutions ex post. But suggesting that policy advisors should ex ante try to consider possible effects on future political equilibria is too ambitious. It has the disadvantage of asking for too much from economic advisors and in addition has the flavour of economist trying to outsmart politicians. Promising too much and then not delivering is a recipe for weakening the influence of economic policy advisors.

## Communication is crucial

Making understandable statements is the most important prerequisite for effective economic policy consulting on all levels. Even the best economic analyses lose their value if politicians do not understand them. Hence, a large part of consulting economists’ work is translating academic findings into everyday language. The challenge for highly specialised economists hereby is translating economic insights, frequently based on technical academic research, into statements, a non-specialist can understand. And this is best achieved by working with simple basic models and a lot of examples—as it is done in good introductory economic textbooks. Indeed, I believe it is no coincidence that of the last nine chief economists of the US government (Chairperson of the Council of Economic Advisors of the President), six have written introductory textbooks in economics; see Table 1.^3^

**Table 1:** CEA heads of Bush II and Obama administrations as textbook authors

Jason Furman	None
Alan Krueger	*Explorations in Economics*
Austan Goolsbee	*Microeconomics*
Christina Romer	None
Edward Lazear	None
Ben Bernanke	*Macroeconomics; Principles of Economics*
Harvey Rosen	*Microeconomics*
Gregory Mankiw	*Principles of Economics; Macroeconomics*
Glenn Hubbard	*Essentials of Economics*

Although these are in most cases highly renowned academic researchers, they were so aware of the great value of clearly explained economic basics that they devoted substantial time and effort to writing such textbooks.

Avoiding complicated reasoning is crucial, but policy advisors should at the same time be differentiated enough in their explanations and avoid populist over-simplification. In my experience, a policy advisor who usually explains in an understandable manner gains enough credibility from the policymaker to be listened to if he occasionally offers necessary differentiation.^4^

The so-called Growth Report, which was written as a basis for federal growth policy in the past decade, in my view is a good example in Swiss economic policy of the added value, a (permitted) simplification can provide.^5^ A glance at the literature shows that growth theory is an exceptionally technical area of economics. And even the use of its most basic models for the explanation of growth policy would not have been digestible for non-specialists. But it is easy to understand that growth can only come from two sources, namely additional hours worked, on the one hand, and additional production per hour worked (= labour productivity), on the other hand. From this simple insight, the decisive conclusion can be drawn that Switzerland—contrary to most EU countries—has already strongly exploited its labour potential and, therefore, additional sustainable growth has to be achieved by increasing productivity. This simple idea was the story the whole growth report was subsequently based on and it proved to be an easily communicable concept in debates with policymakers.

## Economic policy advising in Switzerland

Switzerland has quite a unique political system. The most distinguishing feature is its direct-democratic institutions that strongly shape policymaking in this country. The right to start an initiative for a constitutional change brings frequent votes on far-reaching proposals from outside parliament. However, even more important for the mechanics of how politics works in Switzerland is the referendum. Each proposed change of law can be brought to a public vote by collecting 50,000 signatures, which is quite a small number given the more than 5 million people with a right to vote. This institution has led to a strong process of finding political compromises, that are not challenged by referenda.^6^ One important consequence is that Switzerland since more than half a century is governed by a coalition government that includes all four largest political parties. There is no prime minister and elections do not lead to changes in government—the opposition plays a minor role without ever having the possibility to lead (or even be a member of) the government. And this peculiar system resulting from direct-democratic institutions not only is relevant for the federal level but also for all cantons.

This system very much shapes the role of policy advisors in Switzerland. First and most obviously, due to the frequent votes on specific issues (initiatives and referenda), the need to inform the citizens on (details of) policies plays a much more important role than in purely representative systems. Therefore in this country, public statements by academic economists on economic policy issues potentially have a markedly more direct influence than in systems where the population elects a broad policy platform once every 4 years. And, because this applies at all federal levels, it is especially worthwhile for economists outside the administration to publicly express their opinion on topics relevant to economic policy. Secondly, the fact that there are seven ministries with no prime minister means that majorities have to be found within the administration on all issues. Economic advisors have to be convincing for their colleagues in other ministries in promoting economic arguments in policymaking. Historically, the major economic competences had been split up within the finance and the economics ministries. With the creation of the State Secretariat for Economic Affairs in the last decade, the economists of the economics ministry have been concentrated in one directorate. Since then, this economic policy directorate has assembled a critical mass of economists who now serve as analysts of the economic aspects of all major policies. However, given the need for consensus, these economists can only be effective when constructively and intensively collaborating with the economists of the other ministries. In promoting the government’s growth strategy or in dealing with the policy response to the financial crisis, this collaboration recently proved to be quite effective.

Beyond administration, the economists of business associations and labour unions are particularly influential in Switzerland. This is rooted in the fact that these organisations can always convincingly threaten to fight new legislation by organising a referendum. Government therefore has a strong incentive, to listen to the opinion of these interest groups in shaping legislative proposals. This is done informally by consulting them in the preparation of policies and more formally in the very far-reaching consultation process (the so-called Vernehmlassung) before the government sends a proposal to parliament. Compared to countries with no direct democracies, economic advisors, however, tend to play a smaller role in political parties and in think tanks.

## Concluding remarks

At the beginning of the 1960s, James Tobin shortly served as member of the Council of Economic Advisors for the Kennedy administration. After this assignment ended, in Tobin (1966), he wrote a short piece on advices for economic policy advisors. The following summarises his main points:

- Take the standpoint of the economy as a whole
- Look for ultimate effects, not only at immediate ones
- Worry about silent consumers, not only loud producers
- Examine the mutual consistencies of diverse policies
- Use statistics and history, but handle them with care
- Challenge assumptions that are taken for granted
- Seek alternative solutions with less inefficiencies

In my view, this is still an insightful summary of what is essential to be an effective advisor on economic policy issues.^7^

As I spent almost 20 years in different roles as economic advisor for the Swiss government, let me add to this by summarising in three points what I found to be particularly important in the context of economic policy advice in Switzerland:

Firstly and above all, economists in economic policy consulting act as advocates for the price mechanism. For non-specialists—and hence for most politicians—it is not apparent that a market economy is most efficiently guided by relative prices and not by detailed regulation. We economists have internalised Adam Smith’s invisible hand to a degree that we tend to forget that this brilliant insight is not intuitively evident. Politicians, who want to solve a problem, instinctively prefer organisational and legally verifiable regulation. One of the most central tasks of consulting economists is to point out, as convincingly as possible, alternative ways to achieve goals far more efficiently via undistorted relative prices and market mechanisms instead of detailed planning.

Secondly, and connected, I believe that it is very important for economic advisors to stress the option of “doing nothing”. In the event of economic problems, politicians have a very pronounced tendency to resort to economic policy “actionism”. Quite understandably, they want to be seen taking measures actively. However, if there is no obvious market failure, the most efficient course of action is to do without interventions; doing something then only creates distortions and causes unnecessary uncertainty. Economics ministers who do not always attract attention with new activities do their job especially well in most cases. And, because they will usually be criticised for this approach, it is the economists’ task to provide valid arguments for and to publicly defend efficient doing nothing.

And thirdly, echoing a point also stressed in Tobin’s list, in consulting, economists are responsible that all the economic effects of possible measures are taken into account and not only the most evident ones. Political analyses very often focus on especially influential and well organised groups. However, it is imperative that the impacts on poorly organised (e.g. consumers) or not organised groups (e.g. next generation) are made transparent by economic analyses. Ideally, economists represent the economy as a whole within politics.

All things considered, it is in my opinion crucial for economic arguments to be actively introduced into public debate and the once-in-a-century event in the financial sector has probably led to a strengthening of economic policy consulting in the past years. Although we economists could not fulfil the Queen’s somewhat unrealistic expectations for a prediction of such a crisis, the relatively successful economic policy reactions based on careful analyses of previous crises have demonstrated the benefits of qualified economic policy consulting.

## Footnotes

1 See Staatssekretariat für Wirtschaft (2012) for a more detailed description.

2 For elaborations on this position, see, e.g. Schultze (1982) or Behn (1981).

3 The references are: Krueger & Anderson (2013) Goolsbee et al. (2016), Abel et al. (2008), Frank et al. (2015), Morgan et al. (2009), Mankiw (2015), Mankiw (2017), Hubbard & O’Brian (2012)

4 The tightrope walk for economic policy advisors in communicating with policymakers are well summarised by the following two well-known statements attributed to Albert Einstein: “If you can’t explain it simply, you don’t understand it well enough” and “Everything should be made as simple as possible, but not simpler”

5 See Staatssekretariat für Wirtschaft (2002).

6 See Neidhardt (1970).

7 Other interesting overviews on the major roles of economists in policy making include Ackley (1966), Rivlin (1987), Leman and Nelson (1981) or Hamilton (1992).
